# Analysis of intercellular signal transduction in the tumor microenvironment

**DOI:** 10.1186/1752-0509-7-S3-S5

**Published:** 2013-10-16

**Authors:** Haijun Gong

**Affiliations:** 1 Department of Mathematics and Computer Science, Saint Louis University, St. Louis, MO, 63103 USA

**Keywords:** Tumor microenvironment, signaling pathway, discrete value model, model checking, formal verification

## Abstract

**Background:**

Recent cancer studies revealed, the interaction between pancreatic cancer cells and pancreatic stellate cells is of importance in the cancer progression. The activation of stellate cells is mediated by some growth factors and cytokines secreted by the cancer cells. In turn, the activated stellate cells will synthesize and secrete multiple growth factors to continuously stimulate the growth of surrounding cancer cells through paracrine pathways. The mechanism behind the evolution of stellate cells from quiescent state to a cancer-associated phenotype is still not well understood.

**Results:**

To systematically investigate the interaction between cancer cells and stellate cells, we constructed a multicellular discrete value model, which is composed of several intracellular and intercellular signaling pathways that are frequently mutated in the pancreatic cancer, to study the cell cycle progression and angiogenesis. We, then, introduced and applied a formal verification technique, Symbolic Model Checking, to automatically analyze the cells' proliferation, angiogenesis and apoptosis in the proposed signal transduction model of tumor microenvironment.

**Conclusions:**

Our studies predicted some important temporal logic properties and dynamic behaviors in the pancreatic cancer cells and stellate cells. The verification technique identified several signaling components, including the RAS, RAGE, AKT, IKK, DVL, RB and PTEN, whose mutation or loss of function can promote cell growth and inhibit apoptosis, some of which have been confirmed by existing experiments. Our formal studies demonstrated that, the bidirectional interaction between cancer cells and stellate cells could significantly increase cell proliferation, inhibit apoptosis, induce tumor angiogenesis, and promote cancer metastasis.

## Background

Pancreatic ductal adenocarcinoma (PDAC) is a form of cancer in the pancreatic duct, which is the fourth leading cause of cancer death in the United States, and it has an extremely poor prognosis. The pathological study of PDAC has revealed a number of genetic mutations [1], including the KRAS, CDKN2A, and TP53 genes. A recent global genomic analysis [2] has identified around ten cellular signaling pathways that are frequently altered in pancreatic cancers, including the pathways of Hedgehog, Wnt, Notch, KRAS, apoptosis, TGF-β, cJUN, and G1/S phase transition. In addition, a number of growth factors and cytokines, for example, the Insulin-like growth factor (IGF), Insulin, Hedgehog (Hh), transforming growth factor (TGF-β), and the Advanced Glycation End products (AGEs) are overexpressed in the microenvironment of pancreatic cancer cells, leading to uncontrolled cancer cell proliferation, unorganized angiogenesis and evasion of apoptosis.

Recent experimental studies in pancreatic cancer [3-5] revealed, the interaction between pancreatic cancer cells (PCCs) and pancreatic stellate cells (PSCs, stromal cells of the pancreas) can stimulate cancer progression and tumor angiogenesis (formation of new blood vessels). Pancreatic cancer cells can recruit and activate PSCs to produce and maintain a growth-permissive environment for cancer progression and drug resistance. The activation of PSCs is mediated by several growth factors and cytokines, and many of which are secreted by the pancreatic cancer cells. In turn, the activated PSCs will synthesize and secrete multiple cytokines and growth factors, including Hedgehog and Wnt, through the paracrine and autocrine feedback loops to continuously stimulate cancer cells' growth. These bidirectional interactions [4] will promote cancer progression and unorganized angiogenesis. Besides, PSCs can also secrete a large amount of extracellular matrix (ECM) proteins, which are important components of the fibrous tissue along with stromal cells. Thus, the tumor microenvironment of pancreatic cancer cells and the bidirectional interaction with stellate cells can significantly increase cell proliferation, inhibit apoptosis, induce tumor angiogenesis, and promote cancer metastasis. The mechanism behind the evolution of PSCs from quiescent state to a cancer-associated myofibroblast-like phenotype is still not very clear. Several findings [4,5] have indicated that the pro-angiogenic factor VEGF is of considerable importance in the stellate cell's activation and angiogenesis. To systematically understand the tumor microenvironment and the bidirectional interaction between cancer cells and stellate cells, it is imperative to investigate the intracellular and intercellular signaling pathways that regulate the cell cycle progression and angiogenesis.

Our previous work [6-9] developed Statistical Model Checking and Symbolic Model Checking techniques to study the intracellular signaling pathways in a single cell. Since the pathways implicated in the tumor microenvironment are highly interconnected, to the best of the author's knowledge, no computational multicellular model has been developed to study the interaction between pancreatic cancer cells and stellate cells due to the complexity of networks. In this work, we construct a novel *in silico discrete value model *of multicellular signaling pathways, which are frequently mutated [2] in pancreatic cancers, to study the interaction between PSCs and PCCs. Our 3-cell model is composed of two types of cells: two pancreatic cancer cells (PCCs) and one stellate cell (PSC), which are regulated by the Hedgehog, Wnt, AGE, Rb-E2F, P53, RAS, PI3K, VEGF and IGF signaling pathways. Since the mechanism behind the interaction between PCCs and PSCs is not well understood, our model and analysis will provide some insights into the study of tumor microenvironment and the evolution of stellate cell from a quiescent state to an active state.

In order to formally and automatically analyze the complex network, we introduce a powerful verification technique, called Model Checking [10], which determines whether or not a model (state-transition system) satisfies a desired property expressed in a temporal logic formula. Let *M *be a state-transition system or a model, $S_{0}$ be a set of starting states, and ψ  be a temporal logic formula. The Model Checking problem is to verify that, for all states $s\in S_{0}$, the model *M *satisfies ψ  - denoted by M,s⊨ψ. Model Checker performs an exhaustive search of the state space of the model to verify or falsify the proposed temporal logic formula. Model Checking has been successfully applied to verify hardware systems and digital circuits design. In this work, finally, we apply the Symbolic Model Checking technique to analyze the complex intercellular network of pancreatic cancer cells and stellate cells. A number of important temporal logic and dynamic properties, which specify certain behaviors of regulatory components abstracted from the *in vitro *or *in vivo *experiments in the literature, are proposed to investigate the multicellular signaling pathways in the tumor microenvironment.

## Methods

### Multicellular model of signaling pathways

**Figure 1:**
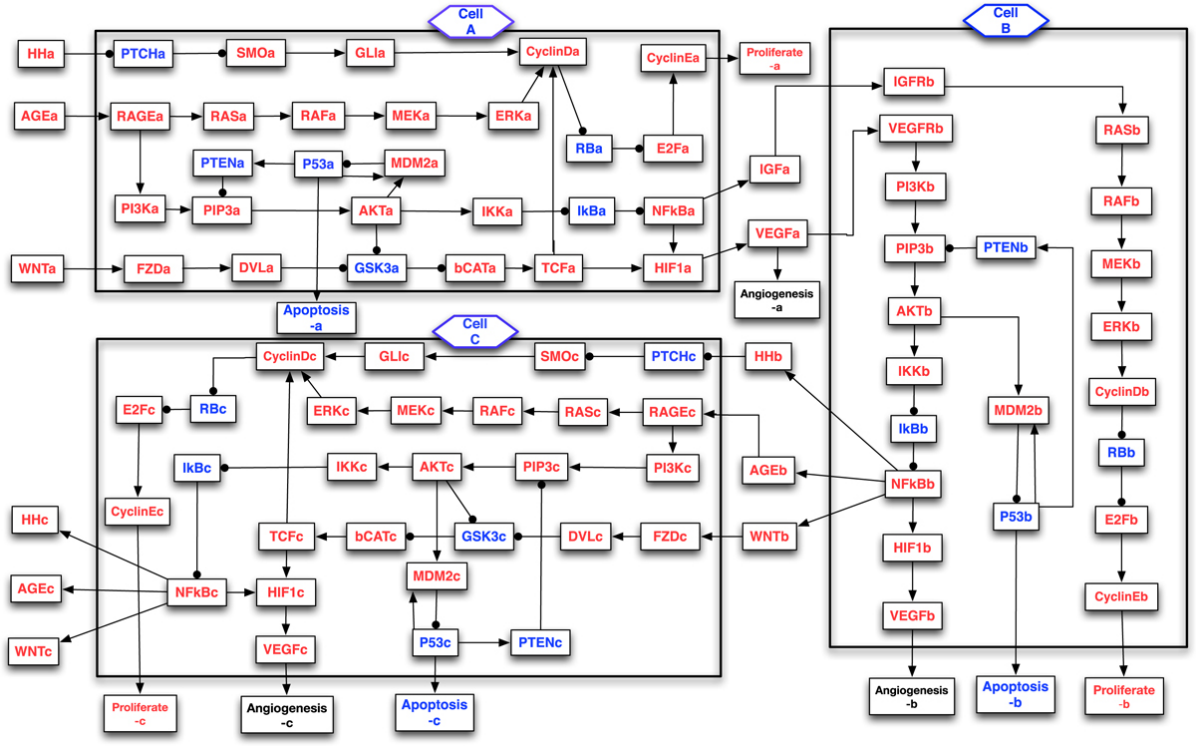
**Multicellular model of signaling pathways in the tumor microenvironment**. Schematic overview of intercellular and intracellular signal transduction in the pancreatic cancer cells and stellate cell. This model is composed of two pancreatic cancer cells (module A and C) and one stellate cell (module B). The suffix (*a, b, c*) in each node represents the cell (A, B, C) that a molecule belongs to. Blue nodes represent tumor-suppressor proteins, red nodes represent oncoproteins or lipids, arrows represent activation, and circle-headed arrows represent deactivation.

Several signaling pathways, including KRAS, Hedgehog, Wnt, Apoptosis, TGF-β, and G1/S phase transition, have been identified as genetically altered in 100% of pancreatic cancers by the global genomic analysis [2]. In addition, many growth factors and cytokines, for example, insulin growth factor (IGF)/Insulin, Hedgehog, WNT and AGEs, can stimulate the growth of cancer cell and secretion of VEGF (a vascular endothelial growth factor), which can promote the evolution of pancreatic stellate cell from quiescent state to active state, and also induce angiogenesis. An extensive literature search was performed to help us construct a multicellular model of signaling pathways, which are composed of the Hedgehog, AGE, WNT-β-Catenin, HIF-1, RAS-ERK, RB-E2F, NFκB, PI3K-P53, IGF, and VEGF pathways in the pancreatic stellate cell and cancer cells. Figure 1 depicts the intercellular model of some signaling pathways implicated in the PCCs and PSCs, some of which have been discussed in our previous single-cell models [6-9]. Our aim is to study the signaling components that regulate the proliferation, apoptosis, and angiogenesis in the pancreatic cancer cells and stellate cells, and bidirectional interactions in the tumor microenvironment using Model Checking technique. In the next sections, we use the symbol →  to denote activation (or promotion), while ⊣  denote inhibition (or repression).

### Intracellular signaling pathways

The paracrine Hedgehog (Hh) signaling is critical for the development of epithelial cells [1,2]. In particular, Hh ligands secreted by the epithelial tumor cells can activate Hh signal transduction in the surrounding stromal cells to stimulates the cell proliferation and contributes to tumorigenesis.

**Hedgehog pathway**: Hh ⊣  PTCH ⊣  SMO →  GLI →  {Hh, CyclinD, ...}. The Hedgehog (Hh) ligand and its receptor Smoothened (SMO) are continuously activated or overexpressed in later-stage pancreatic carcinomas [11], while tumor suppressor protein patched (PTCH) is frequently mutated or loss-of-function, leading to a constitutive activation of Hh pathway. In the quiescent cell without Hh, SMO's activity is inhibited by forming a complex with PTCH. Once Hh binds to PTCH, SMO will be released to activate the GLI (glioma-associated oncogene homologue) to be an active form of transcription factor. The Hh signaling pathway alone is sufficient to drive pancreatic neoplasia [12], and it is known that the activation of the Hh-GLI pathway is associated with tumor proliferation and pancreatic cancer-related fibroblasts [13].

Wnt signaling pathway regulates the processes of angiogenesis and inflammation, and several proteins are genetically altered in most of pancreatic cancers according to the global genomic analysis [2].

**Wnt pathway**: Wnt →  FZD →  DVL ⊣  GSK3β ⊣  β-Catenin →  TCF →  {HIF1, CyclinD, ...}. The canonical WNT pathway is activated by the interaction of Wnt and Frizzled (FZD), leading to the disassembly of Axin-APC-GSK3β complex. Later, the β-Catenin is translocated to the nucleus to activate the TCF-LEF transcription factors [14], promoting the transcription of Cyclin D and HIF-1. However, when the Wnt ligand is absent, β-Catenin is localized in the cytoplasm whose activity will be inhibited by forming a complex with the Axin, APC, and GSK3β [15]. The loss-of-function or continuous activation of some regulatory components in Wnt pathway [16] is responsible for the abnormal vascular development and unorganized angiogenesis.

Recent pancreatic cancer study [17] revealed, the overexpression of the Advanced Glycation End products (AGEs), for example, HMGB1 and its receptor RAGE, is associated with the pancreatic cancer cell's survival. Our previous stochastic and deterministic simulations predicted a dose-dependent p53 and Cyclin E response curve to increasing HMGB1 stimulus in a single cancer cell [6]. AGE pathway regulates the processes of inflammation and angiogenesis.

**AGE-RAS-NFκB pathway**: (1) AGE →  RAGE →  NFκB-pathway; (2) AGE →  RAGE →  RAS-ERK-pathway. The Advanced Glycation End products (AGE), e.g., HMGB1, released by stressed or dying cells, can activate two key signaling pathways [6,7], the RAS pathway: RAS →  RAF →  MEK →  ERK →  CyclinD, which regulates the cell cycle progression through G1 phase; and the NFκB pathway: IKK ⊣  IκB ⊣  NFκB →  {IGF, HIF-1, Hh, Wnt, AGE, ...}. In the resting cell, NFκB is located in the cytoplasm, bound to and inhibited by the tumor suppressor IκB. Once activated by AGE, the IκB kinase (IKK) will phosphorylate and deactivate IκB, leading to the translocation of NFκB into the nucleus to promote the transcription of several genes, including Cyclin D, its inhibitors A20 and IκB (frequently mutated or loss of function) [18,19], and AGEs [20]. The overexpression of NFκB can also stimulate the secretion of VEGF through activating the HIF-1 pathway.

The cell cycle progression is strictly regulated by tens of signaling pathways, and one of the hallmarks is the G1-S phase transition regulated by the RB-E2F-Cyclin E pathway. The global genomic analysis [2] identified several frequently altered regulatory components in this pathway, for example, INK4a and ARF (encoded by CDKN2A) mutations occur in 90% of pancreatic cancers [1].

**G1/S phase transition pathway**: {ERK, TCF, GLI, ...} →  CyclinD ⊣  RB ⊣  E2F →  CyclinE. Some upstream components of the signaling pathway, for example, ERK, TCF and GLI, can activate Cyclin D-CDK4/6 complex which regulates cell cycle progression. In the normal cell, the unphosphorylated RB (a tumor suppressor protein) inhibits E2F's transcription activity by forming RB-E2F complexes. E2F will be activated once its inhibitor RB is phosphorylated and inhibited by Cyclin D. E2F can promote the transcription of Cyclin E [21] and CDK2 complex, which regulates the cell cycle progression from G1 to S phase.

Drug resistance presents a challenge to the treatment of pancreatic cancer. Tumor microenvironment and angiogenesis are two important factors contributing to the drug resistance and cancer development. The environment surrounding a solid tumor is often hypoxic (low oxygen), so that angiogenesis is necessary to provide oxygen and nutrition to support the tumor's growth. The vascular endothelial growth factor (VEGF), a pro-angiogenetic factor whose secretion is mediated by the HIF-1 pathway, can induce angiogenesis. In hypoxic conditions, HIF-1 will be activated and stabilized to regulate the transcription of VEGF [14]. Moreover, it has been reported that the Wnt and NFκB pathways could also upregulate the expression of HIF-1 and VEGF in the cancer cell.

### Intercellualr paracrine signaling pathways

New blood vessels formation (angiogenesis) is one of the key processes in the pancreatic cancer metastasis, and this process is regulated by several pro-angiogenic factors, for example, VEGF, PDGF and HGF. Xu *et al*'s study [5] revealed that the interaction between pancreatic cancer cells (PCCs) and pancreatic stellate cells (PSCs) can stimulate cancer progression and angiogenesis. The activation of PSCs could be mediated by IGF (insulin-like growth factor) and VEGF, which are produced and secreted by pancreatic cancer cells. In turn, the activated PSCs will synthesize and secrete multiple cytokines and growth factors, including Hh, Wnt, AGE, VEGF, etc., to stimulate the growth of neighboring cancer cells and promote angiogenesis through paracrine signaling pathways. In order to systematically investigate the tumor microenvironment, a simple model, which is composed of two PCCs and one PSC, was constructed to investigate the intracellular and intercellular signaling pathways that regulate the cell cycle progression and angiogenesis. In our model, the cells share similar intracellular signaling pathways that were discussed in the last section, and the bidirectional interactions is mediated by VEGF, IGF, WNT, AGE and Hedgehog pathways.

**VEGF-PI3K-NFκB pathway**: (1) VEGF →  VEGFR →  PI3K →  PIP3 →  AKT →  MDM2 →  P53 →  Apoptosis; (2) VEGF →  NFκB-pathway →  {Hh, Wnt, AGE, HIF1, IGF, ...}. VEGF secreted by the cancer cells can bind to its receptor VEGFR on the surrounding stellate cells to activate the PI3K pathway, which will initiate a series of reactions including the phosphorylation of PIP2, AKT, and MDM2, and repression of P53's transcription activity in the nucleus [22]. The tumor-suppressor protein P53, also called the "guardian of the genome", is mutated in more than 50% of pancreatic adenocarcinomas [1]. It is known that P53 can activate the transcription of oncoprotein MDM2 and tumor-suppressor protein PTEN, which is an inhibitor of the AKT pathway and can induce cell cycle arrest. VEGF can also activate the NFκB pathway to promote the transcription and secretion of Hh, Wnt, AGE, HIF1, IGF and VEGF, stimulating the growth of surrounding cancer cells through paracrine feedback loops.

Insulin or Insulin-like growth factor (IGF) pathway can stimulate the growth of pancreatic cancer cells and stellate cells, and inhibit apoptosis through binding and activating its receptor (IGF-1R).

**IGF-RAS pathway**: Insulin/IGF →  IGFR →  RAS →  RAF →  MEK →  ERK →  CyclinD. The overexpressed growth factors, e.g., the Insulin-like growth factor (IGF) and/or Insulin, can activate the RAS protein (K-RAS has a high mutation frequency in pancreatic cancers [1]), leading to the phosphorylation and activation of its downstream proteins RAF, MEK, and ERK [23]. Active ERKs enter the nucleus to phosphorylate the transcription factors myc and promote the expression of the cell cycle regulatory protein Cyclin D, enabling the cell cycle progression through the G1 phase.

### Discrete value model

The interaction between the pancreatic cancer cells and stellate cells is regulated by tens of proteins and crosstalk of different signaling pathways. The traditional computational techniques, including the ordinary differential equation and stochastic simulation methods [6,7], need calculate the reaction rate of each biochemical reaction in the signal transduction. But many parameters are unknown or difficult to be estimated from existing experiments. Our aim is to qualitatively investigate the bidirectional interaction between PCC and PSC in the tumor microenvironment and compare with the experiment. In this work, we develop a discrete value model to describe the expression levels of different signaling components and dynamics of the signaling pathways without introducing any unknown parameters in the biochemical reactions.

In a discrete value model, each node represents a protein or a lipid involved in the signaling pathway. The expression level (state value) of each regulatory component (node) in the pathway can take discrete values at any specific time, for example {0, 1, 2, ..., *n*}, namely, 0 = "not expressed", ..., *n *= "overexpressed". Boolean Network is a special case of discrete value model, which can only take a Boolean value of either ON (1) or OFF (0). The evolution (state update) of each node from time *t *to *t+*1 is described by a discrete state transfer function, which is dependent on the state of the neighboring nodes. In this work, we assume each node can take a value of {0, 1, 2} (it can be extended to *n *discrete values). Similar to our previous work [8,9,24], the neighboring nodes are classified as activators or inhibitors: an activator node can promote or activate the expression of its downstream nodes, while the inhibitor node will inhibit or repress the expression of its downstream nodes. Since this work is the first attempt to investigate the tumor microenvironment using a computational method, for simplicity, in our discrete value model, we assume all the nodes' states are updated synchronously, i.e., the state of each node evolves according to its transfer function at any time step. This assumption has worked well in others' and our previous works on Boolean modeling [8,9,25,26]. Since the biochemical processes in the cells may evolve at different rates, and the synchronous model cannot capture all the information in the cells, we will develop an asynchronous model to investigate the signal transduction in our future work.

The discrete state (update) transfer function for the node $X_{n}$, which is regulated by both activators $A_{i}$ and inhibitors $B_{j}$, in our model can be written as

$$X_{n}\left(t+1\right)=\left\{\begin{array}{c} 2\;\;\;\;if\;\underset{i}{\sum }a_{i}\left(t\right)-\underset{j}{\sum }b_{j}\left(t\right)\geq 2\\ 1\;\;\;\;if\;\underset{i}{\sum }a_{i}\left(t\right)-\underset{j}{\sum }b_{j}\left(t\right)=1\\ X_{n}\left(t\right)\;\;if\;\underset{i}{\sum }a_{i}\left(t\right)-\underset{j}{\sum }b_{j}\left(t\right)=0\\ 0\;\;\;\;if\;\underset{i}{\sum }a_{i}\left(t\right)-\underset{j}{\sum }b_{j}\left(t\right)<0, \end{array}\right)$$

where, $a_{i}$ and $b_{j}$ are the values of the activators $A_{i}$ and inhibitors $B_{j}$ of the node $X_{n}$, respectively. The values "0", "1", and "2" denote the state of "inhibited", "active", and "overexpressed" respectively. For example, PIP3 is activated by PI3K but inhibited by PTEN. At some time step *t*, if PI3K(*t*) = 1, PTEN(*t*) = 0, then, at the next time step, PIP3(*t *+ 1) = 1; if PI3K(*t*) = 2, PTEN(*t*) = 0, then, PIP3(*t *+ 1) = 2; if PI3K(*t*) = 1, PTEN(*t*) = 1, then, PIP3(*t *+ 1) = PIP3(*t*).

In our model, some node is regulated by the inhibitors only. We assume, at time t, if all inhibitors of the node $X_{n}\left(t\right)$ take the value 0, then, at the next time step, $X_{n}\left(t+1\right)=\left\{1,\;2\right\}$ (it can take either 1 or 2, stochastic effect); but if at least one of its inhibitors is active or overexpressed, then, $X_{n}\left(t+1\right)=0$. The state (update) transfer function of these nodes will be written as

$$X_{n}\left(t+1\right)=\left\{\begin{array}{c} 0\;\;\;\;\;\;\;if\;\underset{j}{\sum }b_{j}\left(t\right)\geq 1\\ \left\{1,2\right\}\;\;\;if\;\underset{j}{\sum }b_{j}\left(t\right)=0. \end{array}\right)$$

For example, the proteins RB and E2F, under normal conditions, RB represses E2F's transcription activity by forming RB-E2F complexes. When RB is phosphorylated by Cyclin D, E2F will be activated. So, RB(*t *+ 1) = 0, if CyclinD(*t*) ≥ 1; else, RB(*t *+ 1) = {1, 2}. This assumption is similar to previous work [8,9,25], and consistent with some clinical observation and experimental studies. Many tumor suppressor proteins, including P53, PTEN, INK4a, and ARF, are either mutated or lost in the early or late stages of PDAC. So, they cannot inhibit their downstream oncoproteins; while the oncoproteins, e.g., KRAS and NFκB, are continuously activated or overexpressed, leading to uncontrolled cell growth.

If some node is regulated by the activators only in our model, for example, protein PTEN (frequently mutated or loss of function) whose transcription is regulated by P53 only, we write the transfer functions for these nodes as

$$X_{n}\left(t+1\right)=\left\{\begin{array}{c} 2\;\;\;\;if\;\underset{i}{\sum }a_{i}\left(t\right)\geq 2\\ 1\;\;\;\;if\;\underset{i}{\sum }a_{i}\left(t\right)=1\\ 0\;\;\;\;if\;\underset{i}{\sum }a_{i}\left(t\right)=0. \end{array}\right)$$

However, the output signals, including, Proliferate(*a, b, c*), Apoptosis(*a, b, c*) and Angiogenesis(*a, b, c*), are Boolean variables, which are activated by Cyclin E, P53 and VEGF respectively. In this work, if the corresponding activator's value is greater or equal to 1, the output signal will take a Boolean value of 1 (True); else, it will take a value of 0 (False).

The discrete value model in Figure 1 describes the interactions of different signaling components in the tumor microenvironment, which is composed of *m *= 96 nodes, including 3 control nodes (input signals: Hh, AGE, Wnt), and 9 output nodes (Angiogenesis, Proliferation, Apoptosis in 3 different cells). The structure depicted in Figure 1 represents a "circuit layout" of the cancer-stellate cells model instead of a "state transition" system. Each node in the model is a variable taking *n *possible discrete values (*n *= 2 for 9 output boolean nodes and *n *= 3 for the other 87 nodes in this model), so the number of possible configurations is $n^{m}$ ($3^{87}2^{9}$, around $1.6\times 10^{44}$ possible states). When *n *and *m *are large, the network will have an astronomical amount of possible states. So, it is not realistic to use traditional computational techniques, for example, BooleaNet method [27] and stochastic simulation algorithm [6,7], to analyze such a large network in a fast and effective way. Given a large crosstalk model of signaling pathways, one of our interests is to discover and identify some key cellular components and signal transduction sequences that will drive the system to a pre-specified state (e.g., apoptosis, proliferation or angiogenesis) [8,9,28] at or before a pre-specified time point.

We propose to apply this multi-cellular computational model to investigate the cell-cell interactions of cancer cells with their surrounding microenvironment, in particular, with stellate cells; analyze the paracrine signaling pathways regulating the angiogenesis; identify important proteins that will drive different cells to the "apoptosis", "proliferation" and "angiogenesis" states; simulate the temporal and dynamic behaviors of the cancer cells and stellate cells in various conditions (i.e., drug interference, knockout, or overexpression). To answer these questions, we will introduce the Model Checking and temporal logic properties in the next section.

### Model Checking

Model Checking is a powerful and automatic formal verification technique for finite state transition systems modeled by a Kripke structure [10], which is written as a tuple $M=\left(S,S_{0},R,L\right)$, where, $S_{0}\subseteq S$ is a set of initial states, R⊆S×S is a transition relation between states, and $L:S\to 2^{AP}$ is a function that labels each state *s *with the set of atomic propositions (*AP = *{*p, q, r*, ...}) true in *s*. Given a Kripke structure *M *and a temporal logic formula ψ  expressing some desired property, the Model Checking problem [10] is to find the set of all states in *S *that satisfy ψ , i.e. to compute the set $S_{\psi }=\left\{s\in S|M,s⊨\psi \right\}$. The model *M *satisfies ψ  if $S_{0}\subseteq S_{\psi }$, otherwise, the model checker will output a counterexample (i.e., a sequence of transitions which starts from a state in $S_{0}$) that falsifies the formula ψ .

In the model checking, Computation Tree Logic (CTL) is developed to describe the properties of computation trees. The root of the computation tree corresponds to an initial state and the other nodes on the tree correspond to all possible sequences of state transitions (paths) from the root [10]. A CTL formula is constructed from atomic propositions (AP), Boolean logic connectives (i.e., | (or, ∨ ), & (and, ∧ ), ! (not, ¬), → (implies)), temporal operators and path quantifiers. In the CTL formula, four important temporal operators are used to describe properties on a path [6-9]: **X**p - p will be true in the ne**X**t state of the path; **F**p - p will be true at some state in the **F**uture (eventually) on the path; **G**p - p is **G**lobally true (always, at every state on the path); p **U **q - p holds **U**ntil q holds. In a CTL formula, the operators **X**, **F**, **G**, and **U **must be immediately preceded by a path quantifier **A **- for **A**ll paths, or **E **- there **E**xists a path. Previous work [10] has shown that any CTL formula can be expressed in terms of ¬, ∨ , EX, EU and EG. In this work, we proposed CTL formulas to describe the behaviors or properties of some regulatory components in the signaling pathway. For example, the formula **AG**($MDM2_{active}$ → **AX**$P53_{inhibited}$) means, whenever an MDM2 activation event occurs, it will *always *inhibit P53's transcription activity in the *next *time step.

CTL formulas can be divided into state formulas ψ  and path formulas Φ , and the syntax of the CTL logic is defined as [10]:

$$\psi ::=AP|\psi_{1}\vee \psi_{2}|\neg \psi |E\Phi |A\Phi$$

$$\Phi ::=X\psi \left|F\psi \right|G\psi |\psi_{1}\cup \psi_{2}$$

A path π in a Kripke structure $M=\left(S,S_{0},R,L\right)$ is an infinite sequence of states, that is, $\pi =s_{0},s_{1}$, ..., where $S_{0}$ is an initial state, $s_{i}\in S$ and for every *i *≥ 0, $\left(s_{i},s_{i+1}\right)\in R$. We use $\pi^{i}$ to denote the suffix of π  starting at state $s_{i}$. The semantics of CTL is defined as:

• M,s⊨p iff p∈L(s);

• M,s⊨¬ψ iff M,s⊨ψ does not hold;

• $M,s⊨\psi_{1}\vee \psi_{2}$ iff $M,s⊨\psi_{1}$ or $M,s⊨\psi_{2}$;

• M,s⊨Xψ iff $M,\pi^{1}⊨\psi$;

• M,s⊨Fψ iff there exists a k≥0 such that $M,\pi^{k}⊨\psi$;

• M,s⊨Gψ iff for all k≥0, $M,\pi^{k}⊨\psi$;

• $M,s⊨\psi_{1}\cup \psi_{2}$ iff there exists a k≥0 such that $M,\pi^{k}⊨\psi_{2}$ and for all 0≤j<k, $M,\pi^{j}⊨\psi_{1}$;

• M,s⊨EΦ iff there exists a path π  from *s *such that M,π⊨Φ;

• M,s⊨AΦ iff for every path π  from *s*, M,π⊨Φ;

where M,π⊨Φ means, the path π in *M *satisfies the path formula Φ . More details of CTL semantics can be found in [10].

### Symbolic Model Checking

Model Checking algorithm can automatically and exhaustively search the state transition system to determine, whether or not, a given model *M *satisfies a desired temporal logic formula Φ . The original Model Checking algorithm [10] represents the state transitions *explicitly*. It verifies or falsifies a CTL formula Φ  by recursively labeling the state graph with the sub-formulas of Φ , and then the graph is parsed to compute its truth value in a state for each sub-formula according to the CTL operators and the truth values of its sub-formulas [10,24]. This algorithm could lead to a state explosion problem.

**Figure 2:**
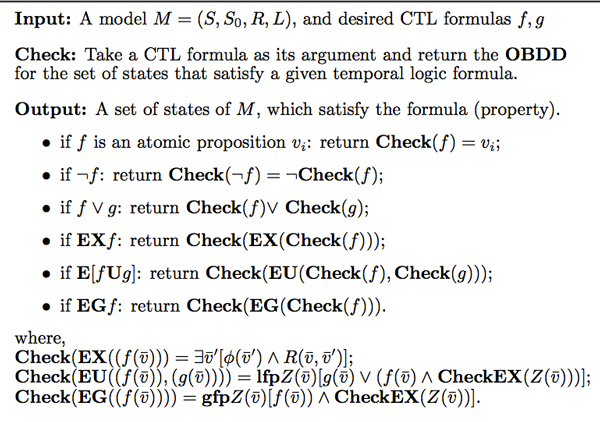
**Symbolic Model Checking algorithm**. Symbolic Model Checking algorithm uses a Boolean function to represent the transition relation between states implicitly, and the Boolean function is encoded by means of an Ordered Binary Decision Diagram (OBDD).

To overcome the state explosion problem, the Symbolic Model Checking algorithm [29] uses a Boolean function to represent the transition relation between states *implicitly*. Moreover, the Boolean function is encoded by means of an Ordered Binary Decision Diagram (OBDD) [30] with a fixed variable ordering and shared sub-graphs. OBDD is an efficient data structure for the representation of Boolean functions. The first CTL model checker based on OBDDs is called the Symbolic Model Verifier (SMV) [10,29], which is an open architecture for model checking. SMV has been widely and successfully applied for the verification of circuit design and hardware systems. Symbolic Model Checking algorithm is reiterated in the Figure 2. In this algorithm, we assume the concurrent system's behavior is determined by *n *boolean state variables $v_{1},v_{2},\ldots ,v_{n}$, and the transition relation is written as $R\left(v_{1},v_{2},\ldots ,v_{n};v_{1}^{\prime },v_{2}^{\prime },\ldots ,v_{n}^{\prime }\right)$, where $\bar{v}=\left(v_{1},v_{2},\ldots ,v_{n}\right)$ and ${\bar{v}}^{\prime }=\left(v_{1}^{\prime },v_{2}^{\prime },\ldots ,v_{n}^{\prime }\right)$ represent the current state and next state respectively, and, **lfp **and **gfp **represent the least and greatest fixpoint respectively. The interested readers can refer to [10,29,30] for details.

During model checking, a model or state transition system (e.g., a signaling pathway) can be described using the SMV language, and a desired cellular behavior or phenomenon can be translated into a CTL formula. Then, SMV model checker will automatically verify or falsify the CTL formula of this model. The output of the verification could be either "true" (property is satisfied) or a counterexample trace showing why the property is false (not satisfied). The complexity of the Symbolic Model Checking algorithm is O(|Ψ | (|S| + |R|)), where |Ψ | is the size of the CTL formula, |S| and |R| are the number of states and transitions respectively [10].

**Figure 3:**
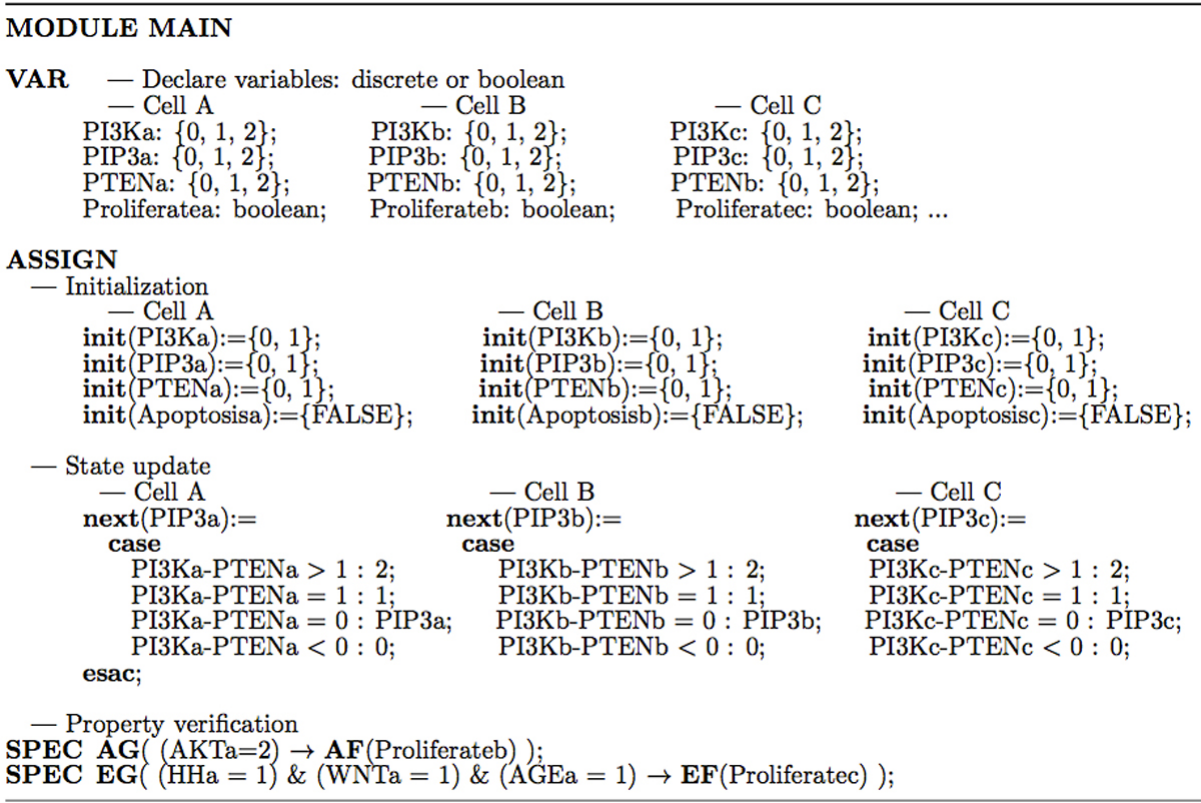
**Illustration of SMV code for the discrete value model**. SMV code can be divided into three parts: variable declaration which is defined by the keyword VAR, initialization and state update which are defined by the keyword ASSIGN, and the property verification is encoded by SPEC.

In Figure 3, we provide part of SMV code to illustrate the procedure to verify a discrete value model of multicellular signaling pathways in the tumor microenvironment. Similar to the single cell Boolean models [8,9], in the SMV code, we use the keyword **VAR **to declare variables first, for example, PI3K*a *can take a discrete value of {0, 1, 2}, and "Proliferate*b*: boolean" means "Proliferate" in cell B takes a Boolean value (0/1). The keyword **ASSIGN **is used to define the initial state (**init**) and state transition (update rules, **next**) of each node. For example, "**init**(PIP3*a*) = {0, 1}" means, the initial value of PIP3 in cell A can be either 0 or 1 (with a probability). The verification of CTL formula is encoded using the "**SPEC**" statement. For example, in Figure 3, **SPEC AG**(AKT*a *= 2 → **AF**(Proliferate*b*)) means, overexpressed AKT in the cancer cell A will finally promote the stellate cell B's proliferation for all paths. The SMV code developed for this discrete value model of signaling pathways is available at [31].

## Results and discussion

The multicellular model of tumor microenvironment illustrated in Figure 1 is composed of two pancreatic cancer cells (PCCs, cell A & C) and one stellate cell (PSC, cell B). The suffix *a, b, c *in each node represent the cell (A, B, C) that a molecule belongs to. For example, "PI3K*a*" represents a "PI3K molecule" in the cancer "cell A". In this model, we use "Proliferate", "Apoptosis" and "Angiogenesis" to represent the fates of the cells, whose initial values are set to be FALSE (0). While, the other nodes, initially, can take a value of either 0 (inhibited) or 1 (active) (a stochastic effect is introduced). We apply SMV model checker to exhaustively and automatically analyze some temporal and dynamic behaviors in the signaling pathways. For simplicity, in this work, we sometimes put (*a, b, c*) behind the temporal logic formulas to represent some regulatory components in the cell A, B and C respectively.

In the cancer studies, we expect to predict the cancer cell's fate in various conditions (i.e., gene knockout, drug interference, loss of function, overexpression), identify key signaling components which play an important role in the tumorigenesis, and explore temporal and dynamic properties in the tumor microenvironment, in a fast and effective way. We first investigate the fates of pancreatic cancer cells (A and C) and stellate cell (B) in a predefined initial condition, that is, all the growth factors are overexpressed in the beginning.

### Cell fate

**Property 1: AF **(Proliferate(*a, b, c*)); and **AF **(VEGF(*a, b, c*) ≥ 1).

Property 1 means, when the proteins Hh, Wnt, AGE (input signals) surrounding the cancer cell A are all overexpressed (i.e., initially HH(*a*) = 2, WNT(*a*) = 2, and AGE(*a*) = 2), the pancreatic cancer cells and stellate cell will finally reach the "Proliferate" state, and VEGF molecules in all three cells (A, B, C) will be in a state of "active" or "overexpressed", for all paths. These two properties are verified to be true. That is, overexpression of some growth factors will stimulate the synthesis and secretion of VEGF from the cancer cell (A), which might activate the stellate cell (B) by the paracrine signaling pathways, promoting the growth of stellate cell B and cancer cell C finally.

**Property 2: **a) **AF **(**!**Apoptosis(*a, b, c*) **&**P53(*a, b, c*) < 1); b) **AF **(Apoptosis(*a, b, c*)).

Property 2 a) tests, for all paths, whether or not, the cancer cells (A, C) and stellate cell (B) will finally reach "Apoptosis" state when Hh, Wnt, and AGE are overexpressed. SMV model checker verified that, all cells cannot reach "Apoptosis" state and the tumor suppressor P53's expression is suppressed (taking a value of 0); while the property b) is falsified (a counterexample trace is provided by SMV). Property 1 and 2 can be summarized as one property which is verified to be true using the SMV model checker:

**Property 3: AF **(P53 < 1 **& !**Apoptosis **&**Angiogenesis **&**Proliferate)(*a, b, c*).

This property means, the cancer cell or stellate cell will reach a state in which apoptosis is inhibited while cell proliferation and angiogenesis are activated. This property is consistent with [5]'s discovery, and explains why the overexpressed growth factors and bidirectional interaction between pancreatic cancer cells and stellate cells can promote cancer progression and angiogenesis, and inhibit the cell's apoptosis.

Next, we will apply the SMV model checker to identify key regulatory components (the most frequently mutated genes or driver genes, *etc*.) and signal transduction sequences that will drive the system to a pre-specified state (e.g., apoptosis, proliferation or angiogenesis) in the tumor microenvironment of pancreatic cancer. We assume the initial values for all nodes, except the output signals, can take a value of either 0 or 1.

### Identification of key oncoproteins

**Property 4: AG**{(RAS(*a*) = {1, 2}) **→ AF**(Proliferate(*a*))};

**Property 5: AG**{(RAS(*a*) = 2) **→ AF**(VEGF = 2 **&**Proliferate **&**Angiogenesis **& !**Apoptosis)(*a, b*)}.

K-RAS mutation occurs in more than 90% of pancreatic cancers [1], especially in the precancerous stage. Constitutively active RAS pathway can stimulate the production of other key proteins during the tumorigenesis. Property 4 verified that, if the oncoprotein RAS in the cancer cell A is active or overexpressed (taking a value of 1 or 2), cell A will finally reach a "Proliferate" state, for all paths. If RAS is mutated or overexpressed (with a value 2) in the cancer cell A, it will stimulate the production and secretion of VEGF, which promotes both cancer cell A and stellate cell B to eventually enter the "Proliferate & Angiogenesis" state, while "Apoptosis" is inhibited. The verified "Property 5" demonstrated that, abating (or turning "off") the signaling function of RAS could provide a rational therapy for pancreatic cancer, and paracrine pathways play an important role in mediating the PSC-PCC interaction in the tumor microenvironment.

Using model checker, besides RAS protein, we can propose similar properties to identify other key oncoproteins whose constitutive activation or mutation in the corresponding signaling pathways will influence the cell's fate. Several oncoproteins, including RAGE, DVL, AKT, and IKK, were verified to play an important role in the tumorigenesis. The following properties were checked.

**Property 6: AG**{(RAGE(*a*) = 2) → **AF**(NFκB = 2 **&**Proliferate **&**Angiogenesis **& !**Apoptosis)(*a, b*)};

**Property 7: AG**{(DVL(*a*) = 2) → **AF**(NFκB = 2 **&**Proliferate **&**Angiogenesis **& !**Apoptosis)(*a, b*)};

**Property 8: AG**{(AKT(*a*) = 2) → **AF**(VEGF = 2 **&** Proliferate **&**Angiogenesis **& !**Apoptosis)(*a, b*)};

**Property 9: AG**{(IKK(*a*) = 2) → **AF**(HIF1 = 2 **&**Proliferate **&** Angiogenesis **& !**Apoptosis)(*a, b*)}.

Property 6 and 7 predicted that, overexpression of RAGE or Dishevelled (DVL) will promote the expression of NFκB in both types of cells. This is consistent with Kang *et al*'s discovery [17], expression of the receptor for advanced glycation endproducts (RAGE) can limit apoptosis and promote pancreatic cancer cell's survival. The oncoprotein AKT and IKK's expression is elevated in many cancers [32,33]. Our previous work [6,7], using stochastic simulation and ordinary differential equation methods, predicted a dose-dependent P53, NFκB and CyclinE response curve to the increase of AKT and IKK. Property 8 and 9, using SMV model checker, revealed that, overexpression of AKT and IKK can increase the production and secretion of VEGF and HIF1 (Hypoxia-inducible factor 1), promote the cancer cell and stellate cell to the "Proliferate & Angiogenesis" state and inhibit "Apoptosis". These properties suggest some possible ways to inhibit tumor growth and promote apoptosis through inhibiting AKT and IKK pathways, e.g., using the AKT kinase inhibitor (such as the drug GSK-690693) and IKK inhibitor (e.g., Manumycin A).

### Identification of key tumor suppressors

The cell cycle progression is regulated by both oncoproteins and tumor suppressors. Next, we apply SMV model checker to identify key tumor suppressors whose activation can promote apoptosis and inhibit proliferation. We analyzed the following properties:

**Property 10: EG**{(RB(*a*) = 2) → **AF**(ERK = 0 **&**Apoptosis **& !**Proliferate **& !**Angiogenesis)(*a, b*)};

**Property 11: EG**{(PTEN(*a*) = 2) → **AF**(CyclinD = 0 **&**Apoptosis **& !**Proliferate **& !**Angiogenesis) (*a, b*)};

**Property 10': AG**{(RB(*a*) = 2) → **AF**(ERK = 0 **&** Apoptosis **& !**Proliferate **& !**Angiogenesis)(*a, b*)};

**Property 11': AG**{(PTEN(*a*) = 2) → **AF**(CyclinD = 0 **&**Apoptosis **& !**Proliferate **& !**Angiogenesis) (*a, b*)}.

Property 10 and 11 were verified to be true, which means, in the RB- or PTEN-treated cells, there EXISTS a path, such that both cancer cell and stellate cell could reach the "Apoptosis" state finally, and the oncoprotein ERK and Cyclin D's expression is repressed. It explained why some single-gene targeted therapies had anti-tumor effects in some pre-clinical studies. However, property 10' and 11' were falsified by the SMV model checker, which means, targeting RB or PTEN in the cancer cell can NOT, for ALL paths, eventually promote the cells to enter a state that "Apoptosis" is ON and "Proliferate & Angiogenesis" are OFF. These properties demonstrate that, the crosstalk between different signaling pathways may be responsible for the pancreatic cancer cell survival even if some pathways are blocked by certain single-gene targeted therapies.

### Necessary checkpoint

**Property 12: !E**{(CyclinD ≤ 1 **&**P53 ≥ 1 **&**HIF1 ≤ 1) **U **(**!**Apoptosis **&**Proliferate **&**Angiogenesis)}(*a, b*);

**Property 12': !E**{**!**(CyclinD >1 ∨  P53 < 1 ∨  HIF1 >1) **U **(!Apoptosis **&**Proliferate **&**Angiogenesis)}(*a, b*);

**Property 13: !E**{VEGF(*a*) ≤ 1 **U **(**!**Apoptosis **&**Proliferate **&**Angiogenesis)(*b*)}.

Here, we want to identify a necessary checkpoint that the pancreatic cancer and stellate cell will go through before they reach a predefined state. A possible checkpoint encoded in the Property 12 and 12' is verified to be true. It is worth to note that, property 12 and 12' are equivalent. And in this property, the operator "**U**" means "until". This formula means, there is no path in which the state "!Apoptosis & Proliferate & Angiogenesis" (state S2) is satisfied without satisfying "CyclinD > 1 ∨  P53 < 1 ∨  HIF1 > 1" (state S1) first. In other words, S1 is a necessary checkpoint for S2. This property demonstrated that, before reaching the cancerous state, the tumor suppressor P53 should have lost functions or been repressed, while oncoproteins Cyclin D or HIF1 are overexpressed or continuously activated in the cells. This property is consistent with existing experimental results that P53 is frequently mutated and Cyclin D is overexpressed in many pancreatic cancers [34]. Property 13 is false, which means, VEGF secreted by the cancer cell is not a checkpoint before the stellate cell reaches proliferation state.

Finally, we apply the SMV model checker to analyze some dynamic behaviors in the multicellular network. Oscillation is an interesting phenomenon in the signaling pathway, which has been studied in the single cell models [6-9] due to the existence of negative feedback loops.

### Dynamic behaviors

**Property 14: AG**{(P53 > 1 → **AF**(MDM2 > 1)) **&**(MDM2 > 1 → **AF**(P53 < 1))}(*a, b, c*);

**Property 15: AG**{HH(*a*) > 1 → **AG**((P53 > 1 → **AF**(MDM2 > 1)) **&**(MDM2 > 1 → **AF**(P53 < 1)))(*a, b, c*)};

**Property 16: AG**{AGE(*a*) > 1 → **AG**((P53 > 1→**AF**(MDM2 > 1)) **&**(MDM2 > 1 → **AF**(P53 < 1)))(*a, b, c*)};

**Property 17: AG**{WNT(*a*) > 1 → **AG**((P53 > 1 → **AF**(MDM2 > 1)) **&**(MDM2 > 1 → **AF**(P53 < 1)))(*a, b, c*)};

**Property 18: AG**{(HH > 1 ∨  WNT > 1 ∨  AGE > 1)(*a*) → **AG**((P53 > 1 → AF(MDM2 ≥ 1)) **&** (MDM2 > 1 → AF(P53 < 1)))(*a, b, c*)}.

Recent experimental study [35] in a single cell observed a dynamic phenomenon of P53 and MDM2, whose expression levels in the nucleus continuously oscillated for more than 72 hours following γ irradiation. This phenomenon was studied in our previous statistical model checking based on stochastic simulations [6,7] and Boolean network models in a single cell in response to HMGB1 stimulus [8]. Property 14 demonstrates that, this phenomenon also exists in the discrete value model of cancer cells and stellate cells due to a self-contained negative feedback loop. Moreover, our multi-cellular model predicts that (Properties 15-18), the external stimulus, for example, overexpression of Wnt, Hedgehog and AGE molecules around the cancer cell, can also induce the oscillation of P53 and MDM2's expression levels in the nucleus in the surrounding stellate and cancer cells. Properties 15-18 were verified by the SMV model checker. Compared with [6,7], the oscillation phenomenon is parameter-independent in our discrete value model using the Symbolic Model Checking method.

## Conclusions

In this work, we developed a discrete value model of multicellular signaling pathways to study the interactions between pancreatic cancer cells and pancreatic stellate cells. The model incorporates several signaling pathways that are frequently mutated in the pancreatic cancer. The powerful Symbolic Model Checking technique is introduced and applied to analyze and validate this model formally. Several interesting temporal logic properties, which encode the cell fate, protein-protein interaction and dynamic behaviors of some regulatory components, are proposed and verified. Compared with our previous statistical model checking work based on stochastic simulations [6,7] and Boolean network method [8,9], the beauty of this technique lies in its flexibility and universality. The signaling components in the model can take any kind of discrete values (3 possible values in this work), and it is easy to be extended to *n *possible values. Without introducing any unknown parameters, the proposed technique has checked up to $10^{44}$ possible states of the multicellular network in tens of minutes, which is not realistic in the traditional simulation methods based on Gillespie's stochastic simulation algorithm and ordinary differential equations. Moreover, the Statistical Model Checking algorithm [6,7] can only verify that a property is true with a probability, and it cannot output a counterexample if some property is not satisfied.

This work identified several genes or proteins, including RAS, RAGE, AKT, DVL, IKK, RB and PTEN, whose mutation or loss of function could promote the cancer cell and stellate cell's proliferation and inhibit apoptosis, leading to uncontrolled growth and unorganized angiogenesis in the future. The verified properties also explained, why certain single-gene targeted therapies, for example, the RB- and PTEN-treatment, might not always inhibit the growth of pancreatic cancer cells, due to the crosstalk of different signaling pathways, even if some pathway is blocked. These properties are either consistent with existing experimental studies, or could be verified or falsified by the future experiments.

We also investigated the dynamic behaviors in the PCCs and PSCs. The expression levels of P53 and MDM2 have been shown to oscillate in a single cell in the previous experimental study and our stochastic simulations [6,7]. This work verified that, in response to external stimulus, the P53-MDM2 network oscillation also exists in the discrete value model of multicellular signaling pathways. Our work revealed, the bidirectional interaction would continuously stimulate the neighboring cell's growth through activating the paracrine signaling pathways, in particular, VEGF pathway. Using Model Checking technique and discrete value model, we can only qualitatively compare with existing experimental discoveries. But formal analysis of this multicellular model still provides valuable information about the interactions between pancreatic cancer cells and stellate cells.

Since the proposed model is only composed of the signaling pathways that are frequently altered in the pancreatic cancer, we are far from capturing all the information in the tumor microenvirnoment, which is, in fact, regulated by tens of signaling pathways and hundreds of proteins. Experimental study [5] found that, the pancreatic stellate cells could secrete a large amount of extracellular matrix (ECM) proteins, which are important components of the fibrous tissue in the pancreatic cancer progression. Since this work attempts to investigate the interaction between PCCs and PSCs for the first time, we only consider the Hedgehog, WNT, AGE, VEGF and IGF proteins secreted by PCCs and PSCs, ECM was not incorporated into our model. A larger network of multicellular signal transduction in the tumor microenvironment will be explored in our future work. Moreover, in this work we assume all the reactions occur synchronously, i.e., the state of each protein (node) is updated at the same time. The synchronous model works well in this work, several interesting properties are consistent with existing experiment. However, biochemical processes may evolve at different rates, sometimes, the synchronous model cannot correctly describe the temporal and dynamic behaviors in the cell. We plan to apply Model Checking to study an asynchronous model in the future work. With the help of Model Checking, a comprehensive understanding of the signaling networks and their crosstalk will help cancer researchers to develop effective multi-gene targeted therapies for the pancreatic cancer patients.

## Competing interests

The authors declare that they have no competing interests.

## Authors' contribution

HG proposed the project, performed the verification and wrote the manuscript.

## Declarations

Publication of this article was funded by the Saint Louis University.

This article has been published as part of *BMC Systems Biology *Volume 7 Supplement 3, 2013: Twelfth International Conference on Bioinformatics (InCoB2013): Systems Biology. The full contents of the supplement are available online at http://www.biomedcentral.com/bmcsystbiol/supplements/7/S3.

## References

[B1] Bardeesy N DePinho RA Pancreatic cancer biology and genetics Nature Reviews Cancer 2002 2 12 897 909 10.1038/nrc949 12459728

[B2] Jones S Zhang X Parsons D Core signaling pathways in human pancreatic cancers revealed by Global genomic analyses Science 2008 321 1801 1806 10.1126/science.1164368 18772397 PMC2848990

[B3] Bachem MG Zhou S Pancreatic stellate cells-role in pancreas cancer Langenbecks Arch Surg 2008 393 891 900 10.1007/s00423-008-0279-5 18204855

[B4] Vonlaufen A Joshi S Pancreatic stellate cells: Partners in crime with pancreatic cancer cells Cancer Res 2008 68 2085 2093 10.1158/0008-5472.CAN-07-2477 18381413

[B5] Xu Z Vonlaufen A Phillips P Role of Pancreatic stellate cells in pancreatic cancer metastasis The American Journal of Pathology 2010 177 5 2585 2596 10.2353/ajpath.2010.090899 20934972 PMC2966814

[B6] Gong H Zuliani P Komuravelli A Faeder JR Clarke EM Analysis and Verification of the HMGB1 Signaling Pathway BMC Bioinformatics 2010 11 Suppl 7 S10 10.1186/1471-2105-11-S7-S10 21106117 PMC2957678

[B7] Gong H Zuliani P Komuravelli A Faeder J Clarke E Computational Modeling and Verification of Signaling Pathways in Cancer Proceedings of Algebraic and Numeric Biology, LNCS 2012 6479

[B8] Gong H Wang Q Zuliani P Lotze MT Faeder JR Clarke EM Symbolic model checking of the Signaling Pathway in pancreatic cancer Proceedings of the International Conference on Bioinformatics and Computational Biology (BICoB) 2011

[B9] Gong H Zuliani P Clarke E Model Checking of a Diabetes-Cancer Model3rd International Symposium on Computational Models for Life Sciences 2011

[B10] Clarke EM Grumberg O Peled DA Model Checking 1999 MIT Press

[B11] Thayer S Di Magliano M Heiser P Hedgehog is an early and late mediator of pancreatic cancer tumorigenesis Nature 2003 425 851 856 10.1038/nature02009 14520413 PMC3688051

[B12] di Magliano M Sekine S Ermilov A Hedgehog/Ras interactions regulate early stages of pancreatic cancer Genes & Development 2006 20 3161 3173 10.1101/gad.1470806 17114586 PMC1635150

[B13] Walter K Omura N Hong SM Griffith M Vincent A Borges M Goggins M Overexpression of Smoothened Activates the Sonic Hedgehog Signaling Pathway in Pancreatic Cancer-Associated Fibroblasts Clinical Cancer Research 2010 16 6 1781 1789 10.1158/1078-0432.CCR-09-1913 20215540 PMC2846609

[B14] Vogelstein B Kinzler K Cancer genes and the pathways they control Nature Medicine 2004 10 789 799 10.1038/nm1087 15286780

[B15] Wodarz A Nusse R Mechanisms of Wnt signaling in development Annu Rev Cell Dev Biol 1998 14 59 88 10.1146/annurev.cellbio.14.1.59 9891778

[B16] Zeng G Germinaro M Micsenyi A Aberrant Wnt/beta-catenin signaling in pancreatic adenocarcinoma Neoplasia 2006 8 279 289 10.1593/neo.05607 16756720 PMC1600679

[B17] Kang R Tang D Schapiro NE Livesey KM The receptor for advanced glycation end products (RAGE) sustains autophagy and limits apoptosis, promoting pancreatic tumor cell survival Cell Death and Differentiation 2009 17 4 666 676 19834494 10.1038/cdd.2009.149 PMC3417122

[B18] van Beijnum JR Buurman WA Griffioen AW Convergence and amplification of toll-like receptor (TLR) and receptor for advanced glycation end products (RAGE) signaling pathways via high mobility group B1 Angiogenesis 2008 11 91 99 10.1007/s10456-008-9093-5 18264787

[B19] Hoffmann A Levchenko A Scott M Baltimore D The IκB-NFκ B Signaling Module: Temporal Control and Selective Gene Activation Science 2002 298 1241 1245 10.1126/science.1071914 12424381

[B20] Kang R Tang D The Receptor for Advanced Glycation End-products (RAGE) Protects Pancreatic Tumor Cells Against Oxidative Injury Antioxidants and Redox Signaling 2011 15 8 2175 2184 10.1089/ars.2010.3378 21126167 PMC3166176

[B21] Yao G Lee TJ Mori S Nevins J You L A bistable Rb-E2F switch underlies the restriction point Nature Cell Biology 2008 10 476 482 10.1038/ncb1711 18364697

[B22] Haupt Y Maya R Kasaz A Oren M Mdm2 promotes the rapid degradation of p53 Nature 1997 387 296 299 10.1038/387296a0 9153395

[B23] Downward J Targeting Ras Signalling Pathways in Cancer Therapy Nature Reviews 2002 3 11 22 12509763 10.1038/nrc969

[B24] Gong H Zuliani P Clarke EM Model checking of a synchronous diabetes-cancer logical network Current Bioinformatics 2013 8 9 15

[B25] Garg A Cara AD Synchronous versus asynchronous modeling of gene regulatory networks Bioinformatics 2008 24 1917 1925 10.1093/bioinformatics/btn336 18614585 PMC2519162

[B26] Mendoza L Xenarios I A method for the generation of standardized qualitative dynamical systems of regulatory networks Theoretial biology and medical modeling 2006 3 13 10.1186/1742-4682-3-13 PMC1440308 16542429

[B27] Albert I Thakar J Li S Zhang R Albert R Boolean network simulations for life scientists Source Code for Biology and Medicine 2008 3 16 10.1186/1751-0473-3-16 19014577 PMC2603008

[B28] Langmead CJ Generalized Queries and Bayesian Statistical Model Checking in Dynamic Bayesian Networks: Application to Personalized Medicine CSB 2009 201 212

[B29] McMillan KL PhD thesis: Symbolic model checking-an approach to the state explosion problem 1992 Carnegie Mellon University

[B30] Bryant R Graph-based algorithms for boolean function manipulation IEEE Tran on Computers 1986 35 8 677 691

[B31] SMV Code http://cs.slu.edu/~gong/PSC.zip

[B32] Altomare D Wang H Skele K Rienzo AD Klein-Szanto A Godwin A Testa J AKT and mTOR phosphorylation is frequently detected in ovarian cancer and can be targeted to disrupt ovarian tumor cell growth Oncogene 2004 23 5853 7 10.1038/sj.onc.1207721 15208673

[B33] Eddy S Guo S Inducible IkBkinase/IkB kinase expression is induced by CK2 and promotes aberrant Nuclear Factor-kB activation in breast cancer cells Cancer Research 2005 65 11375 11383 10.1158/0008-5472.CAN-05-1602 16357145

[B34] Chung DC Brown SB Graeme-Cook F Overexpression of Cyclin D1 Occurs Frequently in Human Pancreatic Endocrine Tumors The Journal of Clinical Endocrinology Metabolism 2000 85 4373 4378 10.1210/jc.85.11.4373 11095482

[B35] Geva-Zatorsky N Rosenfeld N Itzkovitz S Milo R Sigal A Dekel E Yarnitzky T Liron Y Polak P Lahav G Alon U Oscillations and variability in the p53 system Mol Sys Biol 2006 2 2006.0033 10.1038/msb4100068 PMC1681500 16773083

